# Asymmetric Non-Fullerene Small Molecule Acceptor with Unidirectional Non-Fused π-Bridge and Extended Terminal Group for High-Efficiency Organic Solar Cells

**DOI:** 10.3390/ijms231710079

**Published:** 2022-09-03

**Authors:** Kun Wang, Qing Guo, Zengkun Nie, Huiyan Wang, Jingshun Gao, Jianqi Zhang, Linfeng Yu, Xia Guo, Maojie Zhang

**Affiliations:** 1 School of Materials and Chemical Engineering, Zhongyuan University of Technology, Zhengzhou 451191, China; 2 Henan Institute of Advanced Technology, Zhengzhou University, Zhengzhou 450003, China; 3 Laboratory of Advanced Optoelectronic Materials, College of Chemistry, Chemical Engineering and Materials Science, Soochow University, Suzhou 215123, China; 4 CAS Key Laboratory of Nanosystem and Hierarchical Fabrication, CAS Center for Excellence in Nanoscience, National Center for Nanoscience and Technology, Beijing 100190, China

**Keywords:** asymmetric, unidirectional non-fused π-bridge, extended terminal group, non-fullerene small molecule acceptor, organic solar cells

## Abstract

We designed and synthesized an asymmetric non-fullerene small molecule acceptor (NF-SMA) IDT-TNIC with an A–D–π–A structure, based on an indacenodithiophene (IDT) central core, with a unidirectional non-fused alkylthio-thiophene (T) π-bridge, and 2-(3-oxo-2,3-dihydro-1H-cyclopenta[b]naphthalen-1-ylidene)malononitrile (NIC) extended terminal groups. IDT-TNIC molecules still maintain a good coplanar structure, which benefits from the non-covalent conformational locks (NCL) between O···S and S···S. The asymmetric structure increases the molecular dipole moment, and the extended terminal group broadens the absorption of the material, resulting in an excellent photovoltaic performance of IDT-TNIC. The photovoltaic device, based on PBDB-T:IDT-TNIC, exhibits an energetic PCE of 11.32% with a high *V*_oc_ of 0.87 V, high *J*_sc_ of 19.85 mA cm^−2^, and a low energy loss of 0.57 eV. More importantly, IDT-TNICs with asymmetric structures show a superior property compared to symmetric IDT-Ns. The results demonstrate that it is an effectual strategy to enhance the properties of asymmetric A–D–π–A-based NF-SMAs with non-fused NCL π-bridges and extended terminal groups.

## 1. Introduction

Recently, the power conversion efficiency (PCE) of organic solar cells (OSCs) based on non-fullerene (NF) acceptors has exceeded 19%, which leaves researchers full of confidence and expectation for its commercial application [1,2,3,4,5]. In the past 3 years, especially since Y6 was reported in 2019 [6], the development rate of OSCs may be comparable to that in the previous 5–10 years [7]. The rate at which the PCE records continue to be broken is largely due to the rapid development of the symmetrical acceptor–donor–acceptor (A–D–A)-type small molecule acceptors (SMAs) [8,9,10,11,12], which usually consists of an electron-donating unit as the central core, two strong electron-withdrawing units as the terminal groups, and alkyl- or aryl- as side chain groups.

As a new material design concept, the asymmetric NF-SMA strategy has emerged quietly in recent years and achieved impressive results [13,14]. When asymmetric groups or factors appear on a central core, terminal groups or side chain groups, asymmetric NF-SMAs are obtained. Compared with symmetrical A–D–A-type NF-SMAs, asymmetric NF-SMAs retain the structural diversity of the materials and the adjustability of optical properties, and additionally exhibit larger dipole moments and stronger intermolecular interactions than homologous symmetric NF-SMAs [15], thus, they can effectively improve the performance of the OSC device.

Indacenodithiophene (IDT) is one of the most studied and applied central core building units for the constructing of NF-SMAs, because it’s unidirectional, extendable, coplanar structure does not affect the coplanarity and π-electron delocalization, and the side chain group can further adjust the solubility and intermolecular stacking of the materials [9,16,17,18,19]. 

Dipole moments, especially the interfacial dipole moment (donor/acceptor (D/A) interface) and the internal dipole moment of the materials, has a great influence on the charge separation. In 2011, Yu’s group demonstrated that the relatively large internal dipole moment of the polymers can effectively promote the exciton dissociation in the polymer chain [20]. Kim et al. have also shown that sequential fluorination of the polymer backbones increases the dipole moment difference between the ground and excited states [21]. A similar situation has been confirmed in fullerene acceptor [22] and NF-SMAs materials. For example, in 2018, Yang et al. reported a series of asymmetrical NF-SMAs with large dipole moments based on an IDT unit, leading to a higher fill factor (FF) and PCE [23,24]. Various studies have demonstrated that asymmetric SMAs can effectively enhance the properties of materials by extending the IDT backbone on only one side [25,26,27,28,29,30,31,32,33]. Furthermore, other studies have successfully improved material properties through terminal or side chain strategies [14,34,35,36,37,38,39]. However, the long and complicated synthesis hinders the development of unidirectional fused asymmetric SMAs. Multiple studies have shown that a non-fused π-bridge (usually aromatic heterocycles or aromatic heterocycles with side chains) can also expand the conjugate coplanar to broaden the absorption and enhance the intermolecular interaction, which benefits from the non-covalent conformational locks (NCL), that is, the non-covalent bond between O···S, N···S or other atoms [40,41,42,43,44,45].

Recently, our group synthesized an asymmetric A–D–π–A structured SMA with a non-fused NCL π-bridge, namely IDST-4F. Compared with the symmetric ID-4F, the absorption of IDST-4F shows an obvious red shift, the energy level exhibits a slight upshift, whilst the intermolecular interaction also appears enhanced [46], thus, a record PCE was obtained. On the other hand, the intermolecular π–π interaction and the absorption of NF-SMAs can be enhanced by expanding the conjugation area of the terminal group [47,48,49].

Here, we present a strategy that integrates the non-fused NCL π-bridge and extended terminal group to enhance the properties of the NF-SMAs. A new asymmetric NF-SMAs, IDT-TNIC with an indaceno[1,2-b:5,6-b′]dithiophene (IDT) central core, alkylthio-thiophene (T) NCL π-bridge, and 2-(3-oxo-2,3-dihydro-1H-cyclopenta[b]naphthalen-1-ylidene)malononitrile (NIC) terminal groups, was designed and synthesized. PBDB-T with complementary absorption and well-matched energy levels with IDT-TNIC was used as the donor material. The OSCs based on PBDB-T:IDT-TNIC showed an enhanced PCE of 11.32% with an increased open-circuit voltage (*V*_oc_) and reduced energy loss (*E*_loss_) compared to the symmetric analogues.

## 2. Results and Discussion

**Figure 1:**
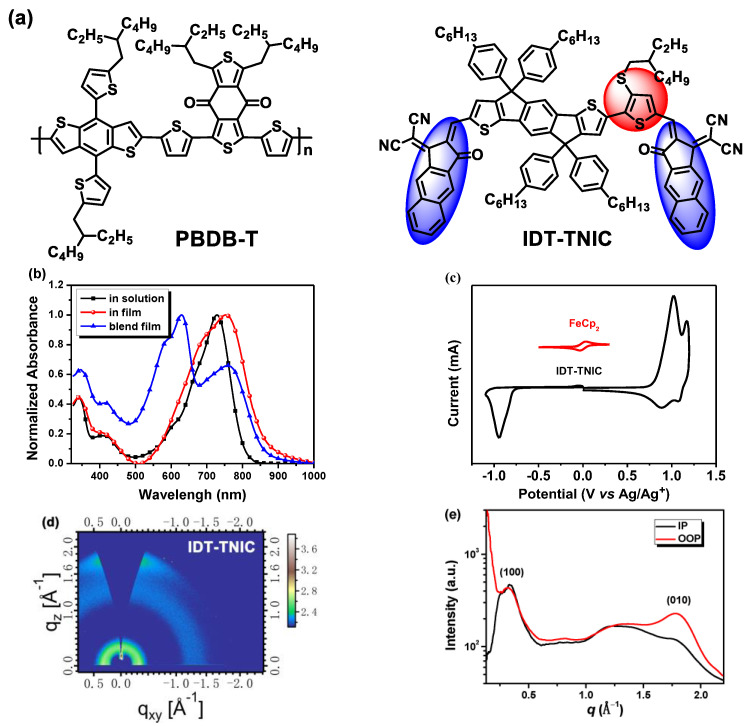
(**a**) Chemical structures of PBDT-T and IDT-TNIC, (**b**) absorption spectra of IDT-TNIC in solution, solid film and PBDT-T:IDT-TNIC blend films, (**c**) cyclic voltammogram of IDT-TNIC, (**d**) the two-dimensional GIWAXS patterns of IDT-TNIC pure film, (**e**) the corresponding in-plane and out-of-plane profiles under optimal condition.

The chemical structures and synthetic routes of IDT-TNIC are shown in Figure 1 and Scheme S1 (in Supplementary Materials). IDT-TNIC shows an excellent thermal stability with a decomposition temperature of 334 °C (Figure S1a), however, no obvious exothermic or endothermic peaks were observed in differential scanning calorimetry (DSC) tests (Figure S1b). X-ray diffraction (XRD) tests show that IDT-TNIC have a weak (100) diffraction peak at 2θ = 4.9° and an indistinct (010) diffraction peak at 2θ = 24.9° (Figure S1c). In grazing incidence wide-angle X-ray scattering (GIWAXS) measurements, IDT-TNIC exhibits a relatively clear (100) diffraction peak along the in-plane (IP) and out-of-plane (OOP) direction (that is, *q*_xy_ and *q*_z_, respectively) at 0.31 Å^−1^ with a *d*-spacing of 20.26 Å (which is arising from the alkyl chain packing), and an obvious (010) π–π stacking peak at *q*_z_ = 1.78 Å^−1^ was also observed with a *d*-spacing of 3.53 Å. This indicates that an IDT-TNIC solid film has a preferred face-on arrangement with the substrate (Figure 1d,e). In addition, IDT-TNIC shows an electron mobility (*μ*_e_) of 2.36 × 10^−4^ cm^2^ V^−1^ s^−1^ that is higher than IDT-N shows using the space-charge-limited-current (SCLC) method (Figure S8 and Table S7). Theoretical calculations results (Figure S2) demonstrate that the π-bridge and the NIC group are almost in a planar configuration, which is formed by the O—S NCL, while the dihedral angle between the IDT and TNIC is about 14.7°. The overall coplanarity of IDT-TNIC is slightly worse than that of IDT-N. IDT-TNIC exhibits a slight upshift in the highest occupied molecular orbital (HOMO) energy level (*E*_HOMO_) and the lowest unoccupied molecular orbital (LUMO) energy level (*E*_LUMO_) compared to IDT-N, and the results are consistent with the experimental results that we will discuss next. The calculated dipole moment (*μ*) is 4.30 Debye (Table S1 and Figure S2).

As shown in Figure 1b, IDT-TNIC in chlorobenzene (CB) solution shows a maximum absorption peak at 727 nm, the IDT-TNIC solid film exhibits a redshift and wider absorption compared to its CB solution, the absorption edge (*λ*_edge_) of IDT-TNIC film was located at 862 nm, and the calculated optical bandgap (*E*_g_^opt^) was 1.44 eV. Moreover, PBDB-T shows a complementary absorption with IDT-TNIC at 300–650 nm, thus, PBDB-T:IDT-TNIC blend film (with a weight ratio of 1:1) shows a wider spectral response from 300 nm to 900 nm. According to the empirical equation [50], the *E*_HOMO_ and *E*_LUMO_ were –5.53 and –3.92 eV, respectively (as shown by the electrochemical cyclic voltammetry (CV) results in Figure 1c), leading to an *E*_HOMO-LUMO_ gaps offset of 1.61 eV. Compared with symmetric IDT-Ns, IDT-TNICs with asymmetric structures exhibit wider absorption and upshifts in frontier orbital energy [51]. The details of the optical and electrochemical comparison parameters of IDT-TNIC and IDT-N are summarized in Table S1 in Supplementary Materials.

**Figure 2:**
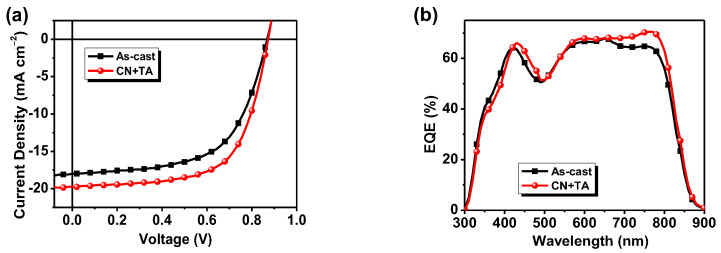
(**a**) *J-V* plots of the PBDT-T:IDT-TNIC-based OSCs (1:1, *w/w*) with 0.75% CN additive, and a TA treatment at 150 °C for 5 min under an illumination of AM 1.5 G, 100 mW cm^−2^. (**b**) EQE curves of the corresponding OSCs.

The photovoltaic performance of IDT-TNIC was investigated using OSC devices with ITO/PEDOT:PSS/PBDB-T:IDT-TNIC/PFN-Br/Al (100 nm) structures. The device optimization process and the corresponding data are listed in Figures S3–S7 and Tables S1–S6. The device performance was optimized by D/A weight ratios, different additives (1,8-Diiodooctane (DIO), N-Methylpyrrolidone, 1-Phenylnaphthalene and 1-Chloronaphthalene (CN)), additive content (0.25%, 0.50%, 0.75% and 1.00%), and then by thermal annealing (TA) temperature (120 °C, 140 °C, 150 °C and 160 °C) and TA time (5 min, 10 min, 15 min, 20 min and 30 min). The PBDB-T:IDT-TNIC-based OSCs achieved an energetic performance of 11.32% with a high *V*_oc_ of 0.87 V, high *J*_sc_ of 19.85 mA cm^−2^ and an FF of 65.9%, when D:A of 1:1, CN as additive (0.75%, *v/v*), and TA treatment at 150 °C for 5 min, while the as-cast device shows a PCE of 10.19% (Figure 2 and Table 1). Significantly, the device exhibits a relatively small energy loss (*E*_loss_) of 0.57 eV, according to the empirical formula *E*_loss_ = *E*_g_−e*V*_oc_, which is smaller than the common values of 0.6–1.0 eV for OSCs. More importantly, the overall performance (especially the *V*_oc_ and *J*_sc_) of OSCs based on asymmetric IDT-TNICs with an NCL π-bridge is better than that of symmetric IDT-N-based devices, as reported in the literature [51]. The enhanced performance of IDT-TNIC is due to the extended coplanar skeleton of the NCL, thus, resulting in a wider absorption spectrum, an upshift in frontier orbital energy, and a stronger intermolecular interaction. Both the devices show broad photo response in the range of 300–850 nm, an EQE value greater than 60% in the range of 400–800 nm, with a maximum EQE value of 70.7% at 768 nm.

**Figure 3:**
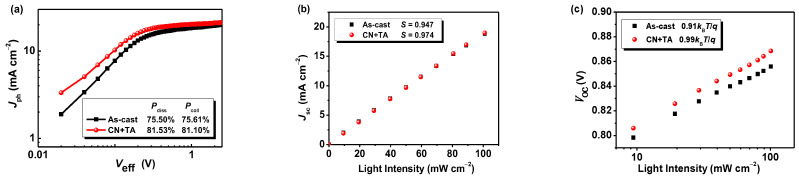
(**a**) *J*_ph_-*V*_eff_ curves; (**b**) *J*_sc_-*P*_light_ fitting lines; and (**c**) *V*_oc_-*P*_light_ fitting lines of the PBDB-T: IDT-TNIC-based devices.

The dependence of *J*_ph_ vs *V*_eff_ can be used to estimate the exciton dissociation efficiency and the charge collection efficiency. Herein, photocurrent density and saturation current are defined as *J*_ph_ and *J*_sat_, respectively [52]. PBDB-T:IDT-TNIC-based optimal devices show a *J*_ph_/*J*_sat_ value of 81.1% in short-circuit and 81.5% for maximum power output conditions (Figure 3a). The recombination loss is smaller than the device as-cast, indicating that the charge transfer is more efficient in optimal devices, thus generating a higher *J*_sc_ and FF. Furthermore, the slope of *V*_oc_ vs ln(*P*_light_) and *J*_ph_∝(*P*_light_)*^S^* were adopted to evaluate the charge recombination mechanism. As shown in Figure 3b, the optimal OSCs exhibit a slightly higher *S* value of 0.974 than as-cast OSCs (0.947), indicating a suppressed bimolecular recombination in optimal OSCs with additive and TA treatment. Moreover, a slope of 0.99 *k_B_T*/*q* was obtained in optimal OSCs, while 0.91 *k_B_T*/*q* for as-cast OSCs, demonstrating that the trap density and trap-assisted recombination can be effectively suppressed in optimal OSCs.

The SCLC method was adopted to measure *μ*_e_ and hole mobility (*μ*_h_) of the pure film and the blend film, to reveal the reason for the high-efficiency of IDT-TNIC-based OSCs (Figure S8 and Table S7). Asymmetric IDT-TNICs show a *μ*_e_ value of 2.36 × 10^−4^ cm^−2^ V^−1^ s^−1^, while the symmetric analogues of IDT-N show a slightly lower *μ*_e_ value of 1.09 × 10^−4^ cm^−2^ V^−1^ s^−1^. When IDT-TNIC blend with PBDB-T, the PBDB-T:IDT-TNIC film shows a more balanced *μ_h_/μ_e_* value (1.14) than PBDB-T:IDT-N film (1.59), which is beneficial to obtain a higher *J*_sc_ and lower *E*_loss_.

**Figure 4:**
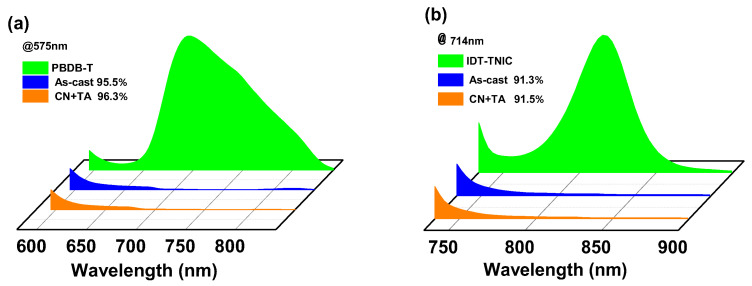
PL spectra of PBDB-T (**a**), IDT-TNIC (**b**) and PBDB-T:IDT-TNIC blend films with excitation wavelengths of 575 nm and 714 nm.

PL quenching efficiencies (*Φ*_PL_) can be used to express the quality of charge transport and exciton dissociation. PBDB-T:IDT-TNIC blend film shows a *Φ*_PL_ values of 96.3% and 91.5% at *λ*_ex_ of 575 nm and 714 nm under optimal conditions, respectively, while the as-cast film shows a slightly lower *Φ*_PL_ values of 95.5% and 91.3%, respectively (Figure 4). The high *Φ*_PL_ values indicate a high-efficient charge transfer between PBDB-T and IDT-TNIC. The results are consistent with the high *J*_sc_ of the devices.

**Figure 5:**
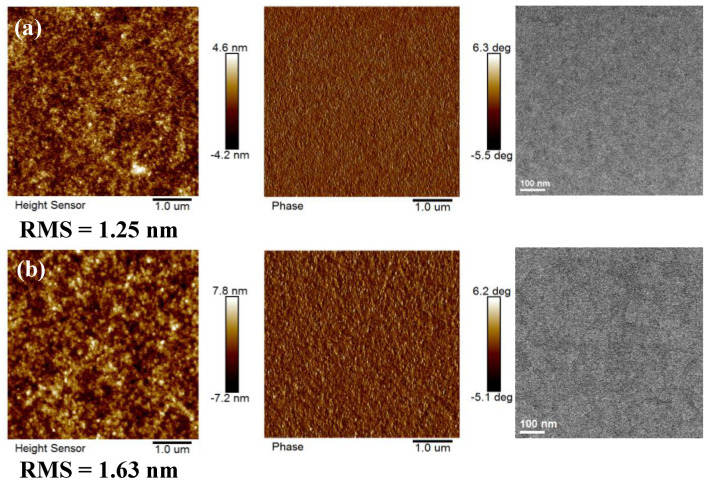
The AFM (left- height image, middle-phase image) and TEM (right) images of optimal blend film of PBDB-T:IDT-TNIC with as-cast (**a**) and CN+TA (**b**).

The active layer morphology directly determines the quality of the device to a great extent. As shown in Figure 5, both as-cast and the optimal film of PBDB-T:IDT-TNIC show a homogeneous surface with a root mean square (RMS) roughness of 1.25 and 1.63 nm, respectively. The transmission electron microscopy (TEM) characterization also demonstrated the active layer possesses an interpenetrating nanofiber structure and suitable phase separation morphology. The good miscibility benefits from the small interaction parameters between PBDB-T and IDT-TNIC (as shown in Figure S8). The results are consistent with the device performance characterization.

Contact angle (CA) measurements were carried out to further study the miscibility and surface tension (*γ*) of donor and acceptor (as shown in Figure S9). We also estimated the interaction parameters (*χ*) between PBDB-T and IDT-TNIC, as the interaction is relevant for phase separation and active layer morphology (the data are summarized in Table S8). The *γ* values of PBDB-T and IDT-TNIC were 34.33 and 43.66 mN m^−1^, respectively. The *χ* value between PBDB-T and IDT-TNIC was 0.56 according to Flory−Huggins model of *χ* = ($\sqrt{\gamma^{D}}$ − $\sqrt{\gamma^{A}}$)^2^ [53,54]. The smaller *χ* value indicates good miscibility between PBDB-T and IDT-TNIC [55,56], which is consistent with the results of the AFM and TEM.

**Figure 6:**
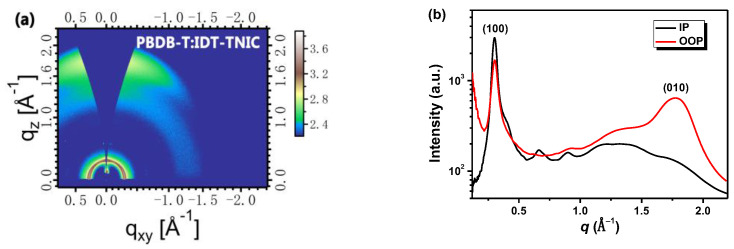
The two-dimensional GIWAXS patterns of PBDB-T:IDT-TNIC blend film (**a**) and the corresponding in-plane and out-of-plane profiles (**b**) under optimal condition.

GIWAXS was adopted to further reveal the crystallinity and molecular orientation of PBDB-T:IDT-TNIC blend film. As shown in Figure 6, distinct and very sharp (100) diffraction peaks appear in IP and OOP directions at 0.30 Å^−1^ with a *d*-spacing of 20.93 Å. Compared with IDT-TNIC pure film, the diffraction peak of PBDB-T:IDT-TNIC blend film at *q*_z_ = 1.77 Å^−1^ with a *d*-spacing of 3.55 Å becomes more prominent, indicating an increased ratio of face-on oriented molecules in the blend film. Furthermore, the blend film shows a larger coherence length (*CL*) of 141.3 Å in the IP direction and 21.74 Å in the OOP direction, according to the Scherrer equation *D*_hkl_ = 2π*K*/∆*q*_hkl_ (*K* is the Scherrer constant, generally = 0.9) [57], the data are summarized in Table S9. The larger CL value from Gaussian fitting (an example is shown in Figure S10) indicates that PBDB-T:IDT-TNIC blend film has a highly ordered molecular arrangement, which is conducive to charge transport.

## 3. Materials and Methods

(1) Synthesis of compound IDT-TNIC

Compound IDT-Th-CHO (0.25 mmol, 297 mg) and NIC (0.75 mmol, 183 mg) were added to a dry two-necked round bottom flask, and then 30 mL CHCl_3_ and 0.5 mL pyridine were added by syringe. The mixture solution was heated to reflux 24 h under argon protection. After cooling to room temperature, the mixture was precipitated into 100 mL methanol, and the solid crude products were collected after suction filtration. The crude product was then purified by column chromatography on silica gel using petroleum ether/CHCl_3_ (*v/v* = 1/1) as the eluent to give a dark, solid product IDT-TNIC (253 mg, 62%). ^1^H NMR (400 MHz, CDCl_3_) δ (ppm): 9.17 (d, *J* = 3.1 Hz, 2H), 8.95 (s, 1H), 8.80 (s, 1H), 8.36 (d, *J* = 10.6 Hz, 2H), 8.20–7.90 (m, 5H), 7.78–7.59 (m, 8H), 7.59–7.49 (m, 1H), 7.24–7.08 (m, 15H), 2.97 (d, *J* = 6.1 Hz, 2H), 2.60 (dd, *J* = 15.2, 7.2 Hz, 8H), 1.72–1.57 (m, 12H), 1.43–1.18 (m, 29H), 0.98–0.78 (m, 18H). MALDI-TOF MS calculated for C_110_H_104_N_4_O_2_S_4_, *m*/*z* = 1641.7076; found [M+H]^+^, 1642.0135. Elemental analyses for CHNS, Found: C, 79.47; H, 6.39; N, 3.47; S, 8.01%; thus the molecular formula C_110_H_104_N_4_O_2_S_4_ required C, 80.45; H, 6.38; N, 3.41; S, 7.81%.

(2) Device fabrication method

The OSC devices were fabricated with the structure of ITO/PEDOT:PSS/PBDB-T:IDT-TNIC/PFN-Br/Al(100 nm). The ITO-coated glass substrate was cleaned with deionized water, acetone, and isopropanol, respectively. Subsequently, the pre-cleaned ITO-coated glass substrate was treated by UV-ozone for 20 min. Then, the PEDOT:PSS were spin-coated onto the ITO-coated glass surface at a spinning rate of 3000 rpm for 30 s, dried at 150 °C for 15 min, then transferred into a nitrogen glove box containing less than 5 ppm oxygen and moisture. The active layer was deposited onto the PEDOT:PSS layer by spin-coating a chlorobenzene solution of PBDB-T:IDT-TNIC with a blend concentration of 25 mg mL^−1^. Then the solution of PFN-Br, which was dissolved in methanol with concentration of 0.5 mg mL^−1^, was spin-coated onto the surface of the active layer-coated ITO with 3000 rpm for 30 s. Finally, 100 nm Al were sequentially evaporated on the active layer in the vacuum chamber under a pressure of ca. 4 × 10^−4^ Pa.

## 4. Conclusions

A new asymmetric NF-SMA IDT-TNIC, with an A–D–π–A structure, was designed and synthesized. Compared with symmetric IDT-Ns, IDT-TNICs show wide absorption, upshifts in energy levels, enhanced intermolecular interactions, and increased dipole moments, thus, leading to an excellent photovoltaic performance. When PBDB-T was used as the donor material, the OSCs based on PBDB-T:IDT-TNIC blend film exhibits an energetic PCE of 11.32% with a high *V*_oc_ of 0.87 V, high *J*_sc_ of 19.85 mA cm^−2^, and a lower energy loss of 0.57 eV. The property of asymmetric IDT-TNIC is better than that of symmetric analogues of IDT-N. The results demonstrate that it is an effectual strategy to enhance the properties of asymmetric A–D–π–A-based NF-SMAs with non-fused NCL π-bridges and extended terminal groups.

## Figures and Tables

**Table 1:** Photovoltaic parameters of the PBDT-T: IDT-TNIC-based OSCs (1:1, *w/w*) with 0.75% CN additive, and a TA treatment at 150 °C for 5 min under an illumination of AM 1.5G, 100 mW cm^−2^.

Active Layer		*V_oc_* (V)	*J_sc_* (mA cm^−2^)	Cal. *J*_sc_^a^ (mA cm^−^^2^)	FF (%)	PCE (PCE_avg_) ^b^ (%)
PBDB-T:IDT-TNIC	As-cast	0.87	18.73	18.10	62.5	10.19 (10.00)
	CN + TA	0.87	19.85	18.86	65.9	11.32 (11.21)
PBDB-T:IDT-N	CN + TA	0.79	15.88	--	71.9	9.0 [51]
PBDB-TF:IDT-N	DIO + TA	0.946	16.58	16.02	78.0	12.2 [49]

^a^ Integral *J_sc_* from EQE curves. ^b^ The average values were collected from 4 devices.

## Data Availability

The data presented in this study are available on request from the corresponding author.

## Supplementary Materials

The supporting information can be downloaded at: https://www.mdpi.com/article/10.3390/ijms231710079/s1. References [51,53,54,58,59] are cited in the supplementary materials.
