# Supplementary material for: Asymmetric Non-Fullerene Small Molecule Acceptor with Unidirectional Non-Fused π-Bridge and Extended Terminal Group for High-Efficiency Organic Solar Cells
Source: Int J Mol Sci. 2022 Sep 3;23(17):10079. doi: 10.3390/ijms231710079 (PMC9456090; doi:10.3390/ijms231710079)
Supplement: Supplementary file 1 [file ijms-23-10079-s001.zip › ijms-1889313-supplementary.pdf]

Supporting Information

Asymmetric Non-fullerene Small Molecule Acceptor with Unidirectional Non-fused π-bridge and Extended Terminal Group for High-efficiency Organic Solar Cells

Kun Wang [1,,†], Qing Guo [2,3,†], Zengkun Nie [1], Huiyan Wang [1,2], Jingshun Gao [1], Jianqi Zhang [4], Linfeng Yu [3], Xia Guo [3,*] and Maojie Zhang [3]*

[1] School of Materials and Chemical Engineering, Zhongyuan University of Technology, Zhengzhou 451191, China

[2] Henan Institute of Advanced Technology, Zhengzhou University, Zhengzhou 450003, China.

[3] Laboratory of Advanced Optoelectronic Materials, College of Chemistry, Chemical Engineering and Materials Science, Soochow University, Suzhou 215123, China

[4] CAS Key Laboratory of Nanosystem and Hierarchical Fabrication, CAS Center for Excellence in Nanoscience, National Center for Nanoscience and Technology, Beijing 100190, China

* Correspondence: kwang@zut.edu.cn (K.W.); guoxia@suda.edu.cn (X.G.)

† Kun Wang and Qing Guo contributed equally to this work

Experimental section

Materials.

All chemicals and solvents were reagent grades and purchased from Alfa Aesar, J&K, and Aldrich, respectively. IDT-Sn [1], IDT-Th [2] and IDT-Th-CHO [2], was synthesized according to reported before. The molecular structure and synthetic route of **IDT-TNIC** is shown in **Scheme S1**, the detailed as follows:

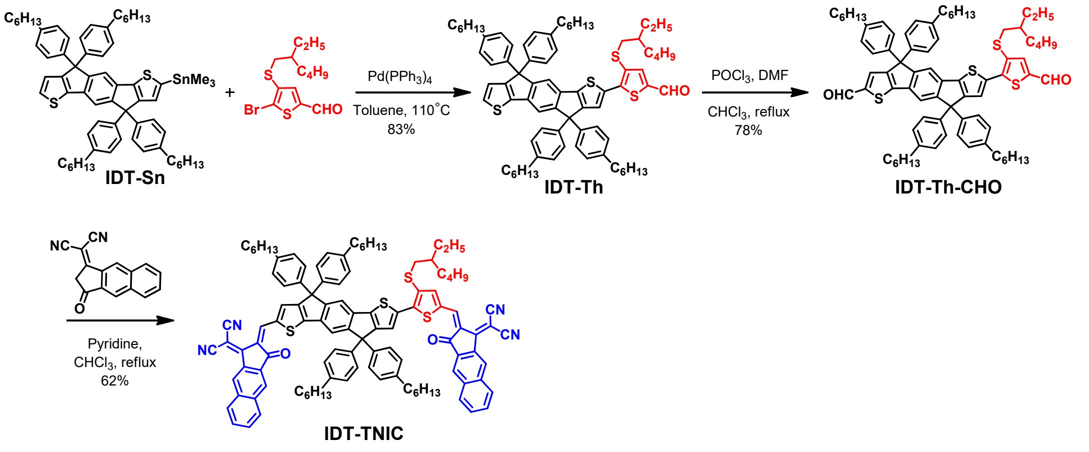

Scheme S1. The synthetic route and molecular structure of **IDT-TNIC**.

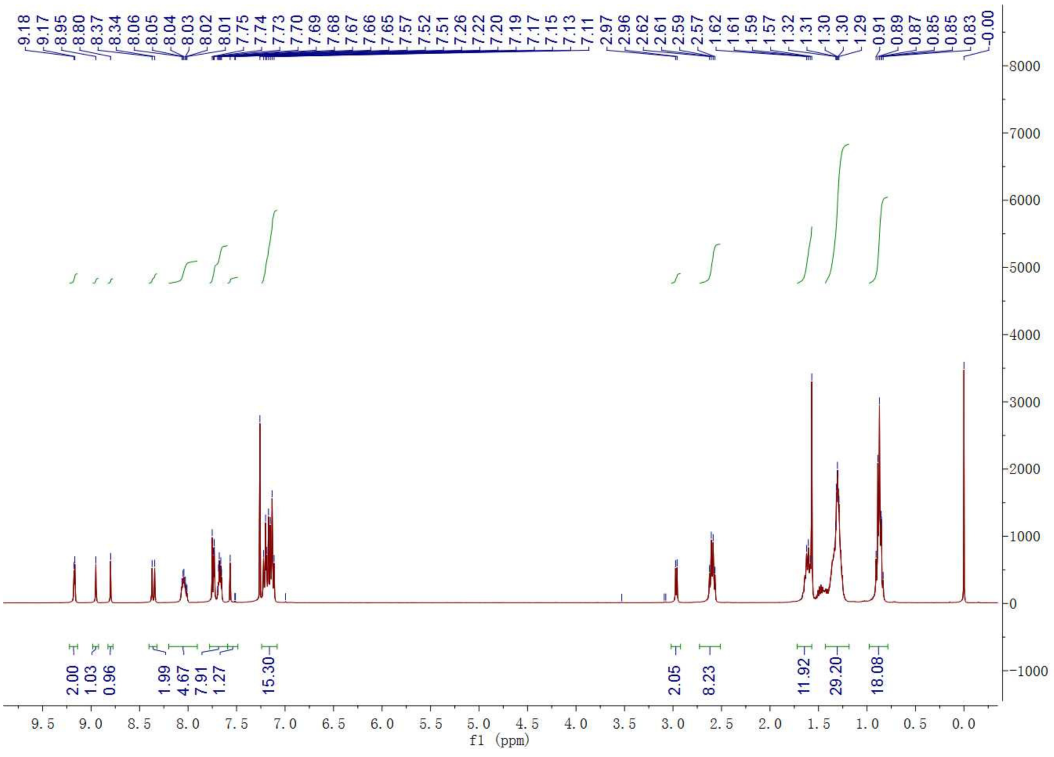

^{1}H NMR of **IDT-TNIC** in $CDCl_3$.

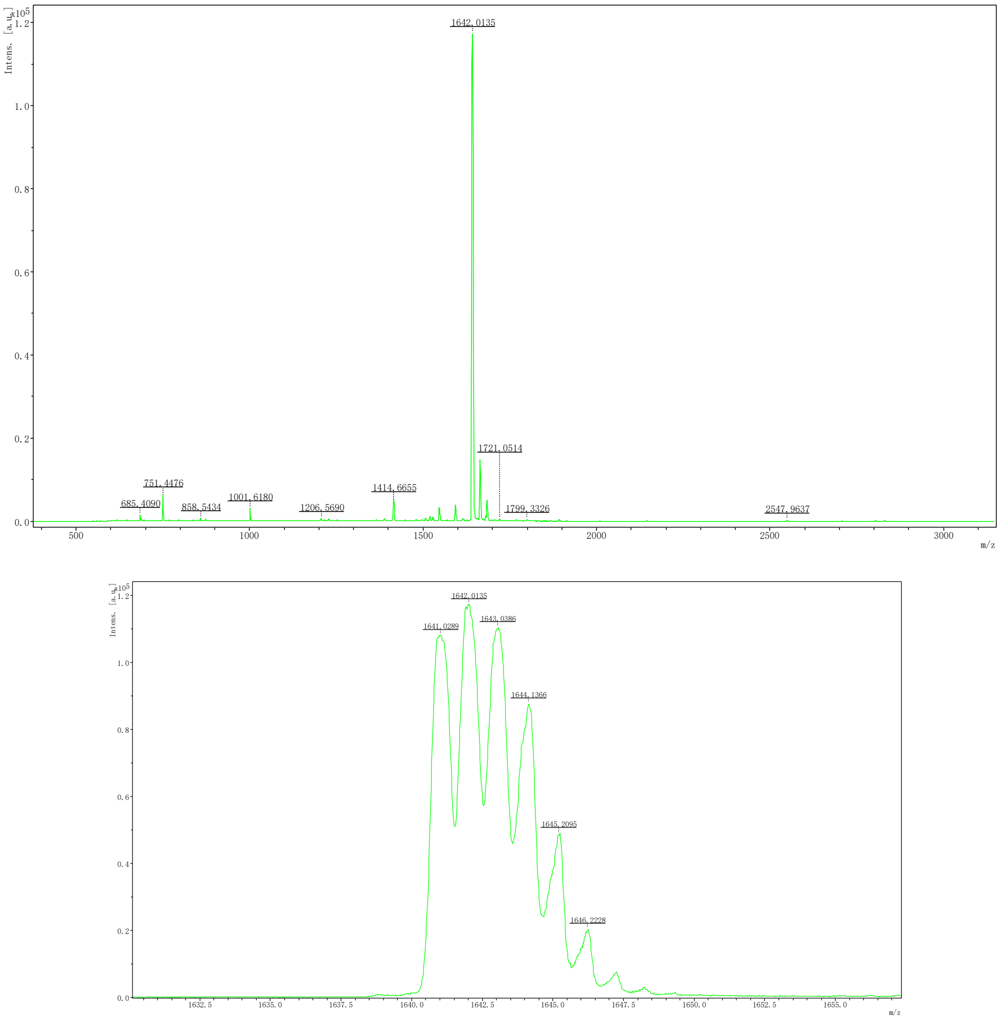

MALDI-TOF of **IDT-TNIC**

Instruments and measurements

^{1}H NMR and ^{13}C NMR were measured in $CDCl_3$ on Bruker AV 400 MHz FT-NMR spectrometer. MALDI-TOF was measured on ultrafleXtreme™ Mass spectrometer from Bruker Daltonics. UV-Vis absorption spectra were measured by an Agilent Carry-5000 UV-Vis spectrophotometer. The solubility of PBSF-A12, PBSF-D12 and PBDB-T-SF in different solution were calculated according to the method reported in the literature. [4] Electrochemical cyclic voltammetry (CV) was performed on a Zahner Zennium IM6 electrochemical workstation with a three-electrode system in 0.1 mol L^{-1} Bu_4NPF_6 acetonitrile solutions at a scan rate of 50 mV s^{-1}. Elemental analysis was carried out on a flash EA1112 analyzer. The molecular weight of the polymer was measured by the GPC method with polystyrene as the standard and 1,2,4-tricholorobenzene as the solvent at 160 °C using Agilent Technologies

PL-GPC220. Thermogravimetric analysis (TGA) was measured on Discovery TGA from TA Instruments Inc. at a heating rate of 10 °C min^{-1} under a nitrogen atmosphere. Differential scanning calorimetry (DSC) was performed on a TA DSC Q-200 at a scan rate of 10 °C min^{-1} under nitrogen atmosphere. X-Ray Diffraction (XRD) was performed on a X'Pert-ProMRD. Photoluminescence (PL) spectra were performed on an Edinburgh Instrument FLS 980. The atomic force microscopy (AFM) measurement was carried out on a Dimension 3100 (Veeco) Atomic Force Microscope in the tapping mode. Transmission electron microscopy (TEM) was performed on a Tecnai G2 F20 S-TWIN instrument at 200 kV accelerating voltage. The GWAXS measurements were performed at beamline 7.3.3 at the Advanced Light Source (ALS). The current-voltage (*J*-*V*) characteristics of the devices were measured on a Keithley 2450 Source Measure Unit. The power conversion efficiency of the OSCs was measured under an illumination of AM 1.5G (100 mW cm^{-2}) using a SS-F5-3A (Enli Technology Co. Ltd.) solar simulator (AAA grade, 50 mm x 50 mm photobeam size). The EQE was measured by Solar Cell Spectral Response Measurement System QE-R3011 (Enli Technololy Co. Ltd.). The light intensity at each wavelength was calibrated with a standard single-crystal Si photovoltaic cell.

Mobility measurement

The mobility was measured by the space charge limited current (SCLC) method by a hole-only device with a structure of ITO/PEDOT:PSS/PBDB-T or PBDB-T:**IDT-TNIC**/MnO_3(10 nm)/Al(100 nm) or an electron-only device with a structure of ITO/ZnO-NP/PBDB-T:**IDT-TNIC** or **IDT-TNIC** /PFN-Br/Al(100 nm) and estimated through the Mott-Gurney equation. For the hole-only devices, SCLC is described by $J \cong (9/8)\ \varepsilon\varepsilon_0\mu_0 V^2 \exp(0.89\sqrt{V/E_0L})/L^3$, Where ε is the dielectric constant of PM6:Acceptors, ε_0 is the permittivity of the vacuum, μ_0 is the zero-field mobility, E_0 is the characteristic field, J is the current density, L is the thickness of the films, $V = V_{appl} - V_{bi}$, V_{appl} is the applied potential, and V_{bi} is the built-in potential which results from the difference in the work function of the anode and the cathode (in this device structure, V_{bi} = 0.2 V). For the electron-only devices, SCLC is

described by $J = \frac{8}{9}\varepsilon_r\varepsilon_0\mu_e\frac{V^2}{L^3}$, where J is the current density, ε_r is the dielectric constant of IDT-TNIC, ε_0 is the permittivity of the vacuum, L is the thickness of the blend film, $V = V_{appl} - V_{bi}$, V_{appl} is the applied potential, and V_{bi} is the built-in potential which results from the difference in the work function of the anode and the cathode (in this device structure, $V_{bi} = 0$ V).

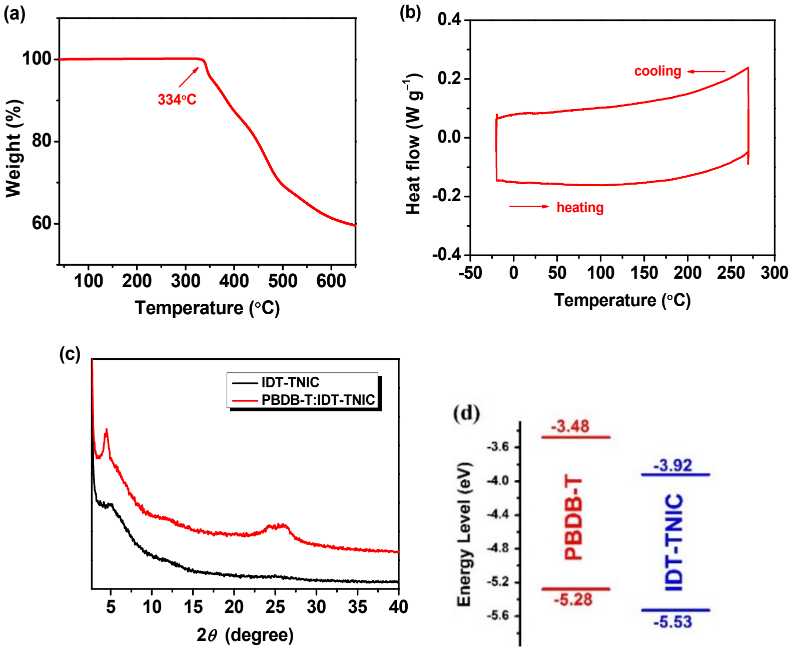

Figure S1. (a) TGA and (b) DSC of **IDT-TNIC**, (c) XRD of **IDT-TNIC** pure film and PBDB-T:**IDT-TNIC** blend film, (d) Energy level diagram of PBDT-T and **IDT-TNIC**.

Table S1. Optical and electrochemical properties of **IDT-TNIC**.

Material	UV-vis in solution		UV-vis in solid film			CV			Dipole moment (Debye) [b]
	λ_{max} (nm)	λ_{edge} (nm)	λ_{max} (nm)	λ_{edge} (nm)	E_g^{opt} (eV)	HOMO (eV)	LUMO (eV)	E_g^{cv} (eV)	
**IDT-TNIC**	727	809	755	862	1.44	−5.53	−3.92	1.61	4.30
**IDT-N**[a]	687	725	727	784	1.58	−5.74	−4.08	1.66	0.00 [3]

[a] Reported in literature. [b] Obtained from gaussian theoretical simulation

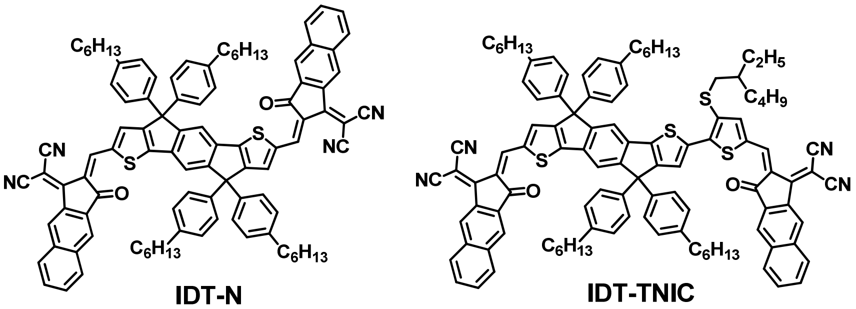

IDT-N **IDT-TNIC**

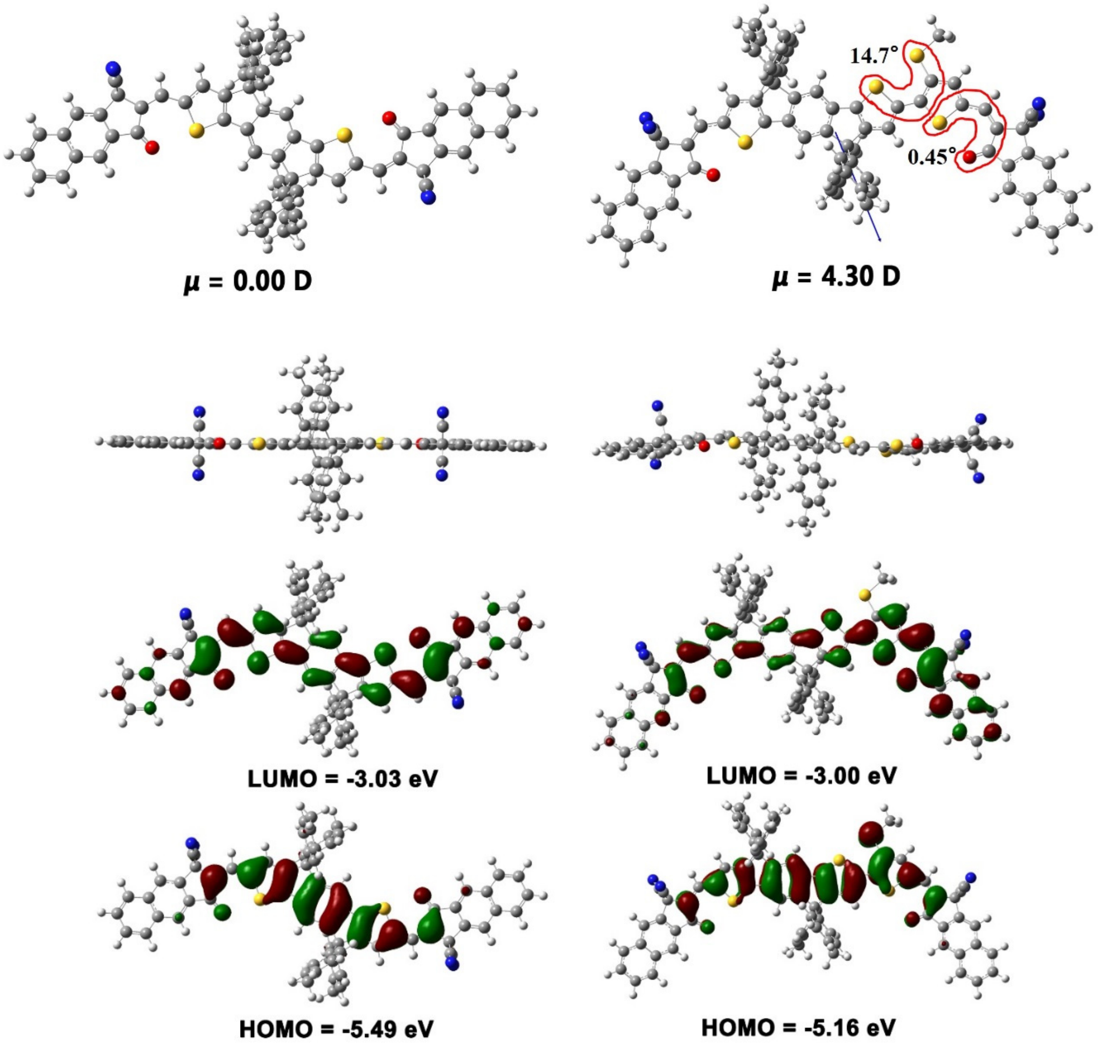

Figure S2. Chemical structures of IDT-N (left) and **IDT-TNIC** (right), and the corresponding molecular conformations and the electron cloud distribution of frontier molecular orbital via DFT-based theoretical calculations at the B3LYP/6-31G(d,p) level. (All side chains are simplified as the methyl groups.)

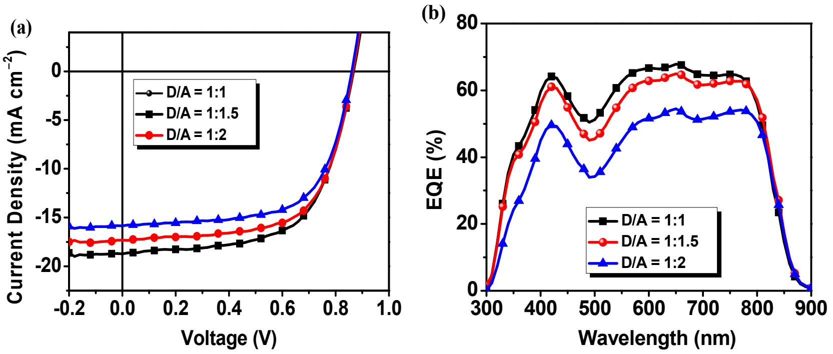

Figure S3. (a) *J-V* plots of PBDT-T:**IDT-TNIC**-based OSCs with different D/A under the illumination of AM 1.5G, 100 mW cm^{-2}, (b) The corresponding EQE curves of the OSCs.

Table S2. Photovoltaic parameters of PBDT-T:**IDT-TNIC**-based OSCs with different D/A under the illumination of AM 1.5G, 100 mW cm^{-2}.

PBDB-T:IDT-TNIC	V_{oc} (V)	J_{sc} (mA cm^{-2})	Cal. J_{sc} (mA cm^{-2})	FF (%)	PCE (%)
1:1	0.868	18.73	18.10	62.5	10.19
1:1.5	0.865	17.32	17.30	64.8	9.72
1:2	0.861	15.86	14.27	65.0	8.87

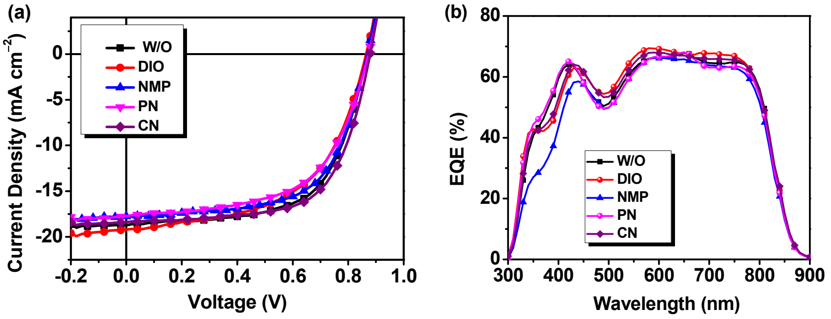

Figure S4. (a) *J-V* plots of PBDT-T:**IDT-TNIC**-based OSCs (1:1, w/w) with different additive under the illumination of AM 1.5G, 100 mW cm^{-2}, (b) The corresponding EQE curves of the OSCs.

Table S3. Photovoltaic parameters of PBDT-T:**IDT-TNIC**-based OSCs (1:1, w/w) with different additive under the illumination of AM 1.5G, 100 mW cm^{-2}.

PBDB-T:IDT-TNIC	V_{oc} (V)	J_{sc} (mA cm^{-2})	Cal. J_{sc} (mA cm^{-2})	FF (%)	PCE (%)
W/O	0.863	18.84	18.10	61.6	10.19
DIO [a)]	0.865	19.20	18.54	55.2	9.32
NMP [b)]	0.869	17.76	17.32	62.3	9.79
PN [c)]	0.873	17.76	17.85	58.0	9.16
CN [d)]	0.879	18.41	18.34	64.6	10.46

[a)] DIO is an abbreviation of 1,8-Diiodooctane. [b)] NMP is an abbreviation of N-Methylpyrrolidone. [c)] PN is an abbreviation of 1-Phenylnaphthalene. [d)] CN is an abbreviation of 1-Chloronaphthalene.

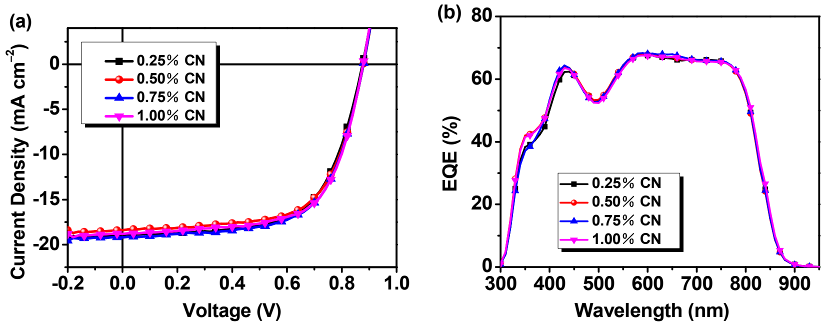

Figure S5. (a) *J-V* plots of PBDT-T:**IDT-TNIC**-based OSCs (1:1, w/w) with different CN contents under the illumination of AM 1.5G, 100 mW cm^{-2}, (b) The corresponding EQE curves of the OSCs.

Table S4. Photovoltaic parameters of PBDT-T:**IDT-TNIC**-based OSCs (1:1, w/w) with different CN contents under the illumination of AM 1.5G, 100 mW cm^{-2}.

PBDB-T:IDT-TNIC	V_{oc} (V)	J_{sc} (mA cm^{-2})	Cal. J_{sc} (mA cm^{-2})	FF (%)	PCE (%)
0.25% CN	0.876	18.98	18.25	63.6	10.58
0.50% CN	0.879	18.41	18.34	64.6	10.46
0.75% CN	0.879	19.20	18.37	64.2	10.84
1.00% CN	0.878	18.81	18.35	65.3	10.79

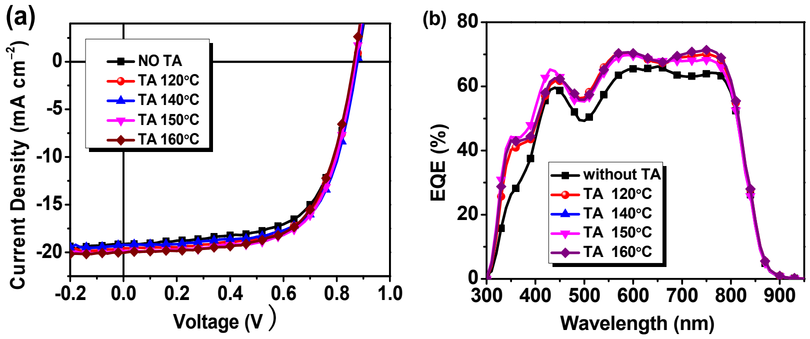

Figure S6. (a) *J-V* plots of PBDT-T:**IDT-TNIC**-based OSCs (1:1, w/w) with different TA temperature for 10 min, 0.75% CN as additive under the illumination of AM 1.5G, 100 mW cm^{-2}, (b) The corresponding EQE curves of the OSCs.

Table S5. Photovoltaic parameters of PBDT-T:**IDT-TNIC**-based OSCs (1:1, w/w) with different TA temperature under the illumination of AM 1.5G, 100 mW cm^{-2}.

PBDB-T:IDT-TNIC		V_{oc} (V)	J_{sc} (mA cm^{-2})	Cal. J_{sc} (mA cm^{-2})	FF (%)	PCE (%)
0.75% CN, TA 10min	NO TA	0.879	19.20	17.58	64.2	10.84
	TA 120 °C	0.874	19.56	19.10	64.9	11.04
	TA 140 °C	0.878	19.21	18.77	66.9	11.21
	TA 150 °C	0.875	19.23	18.93	66.3	11.22
	TA 160 °C	0.865	20.04	19.25	64.2	11.06

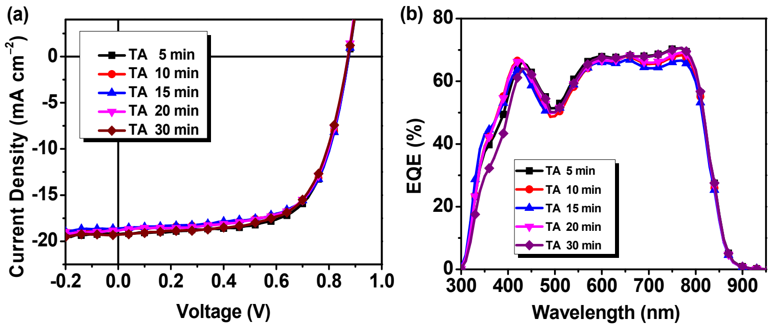

Figure S7. (a) *J-V* plots of PBDT-T:**IDT-TNIC**-based OSCs (1:1, w/w) with different TA time, 0.75% CN as additive and TA treatment at 150 °C under the illumination of AM 1.5G, 100 mW cm^{-2}, (b) The corresponding EQE curves of the OSCs.

Table S6. Photovoltaic parameters of PBDT-T:**IDT-TNIC**-based OSCs (1:1, w/w) with different TA time, 0.75% CN as additive and TA treatment at 150 °C under the illumination of AM 1.5G,

100 mW cm^{-2}.

PBDB-T:IDT-TNIC		V_{oc} (V)	J_{sc} (mA cm^{-2})	Cal. J_{sc} (mA cm^{-2})	FF (%)	PCE (%)
0.75% CN TA 150 °C	5 min	0.871	19.85	18.86	65.9	11.32
	10 min	0.875	19.23	18.41	66.3	11.22
	15 min	0.875	18.62	18.14	67.0	10.97
	20 min	0.872	18.84	18.57	66.2	10.93
	30 min	0.873	19.27	18.63	65.0	10.99

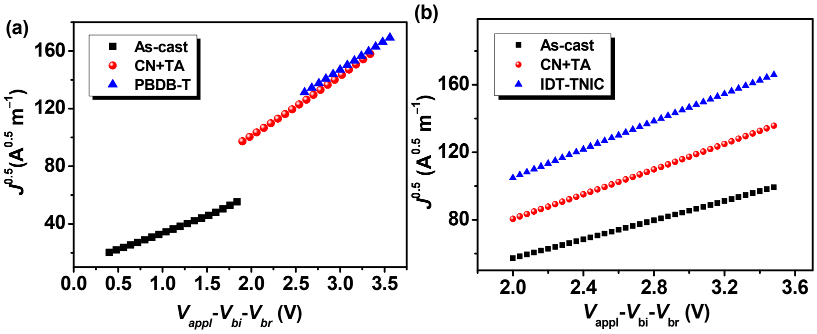

Figure S8. The $J^{0.5}$-V plots for calculations of the hole-only (a) and electron-only (b) mobilities of PBDB-T:**IDT-TNIC**-based devices.

Table S7. Hole and electron mobilities of PBDB-T:**IDT-TNIC**-based devices measured by the SCLC method.

Materials	Conditions	μ_h (cm^{-2} V^{-1} s^{-1})	μ_e (cm^{-2} V^{-1} s^{-1})	μ_h /μ_e
PBDB-T	Pure film	3.91×10^{-4}	--	--
**IDT-TNIC**	Pure film	--	2.36×10^{-4}	--
PBDB-T:**IDT-TNIC**	As-cast	2.30×10^{-4}	1.73×10^{-4}	1.33
PBDB-T:**IDT-TNIC**	CN+TA	3.63×10^{-4}	3.19×10^{-4}	1.14
IDT-N [2]	Pure film	--	1.09×10^{-4}	--
PBDB-T:IDT-N [2]	CN+TA	4.57×10^{-4}	2.87×10^{-4}	1.59

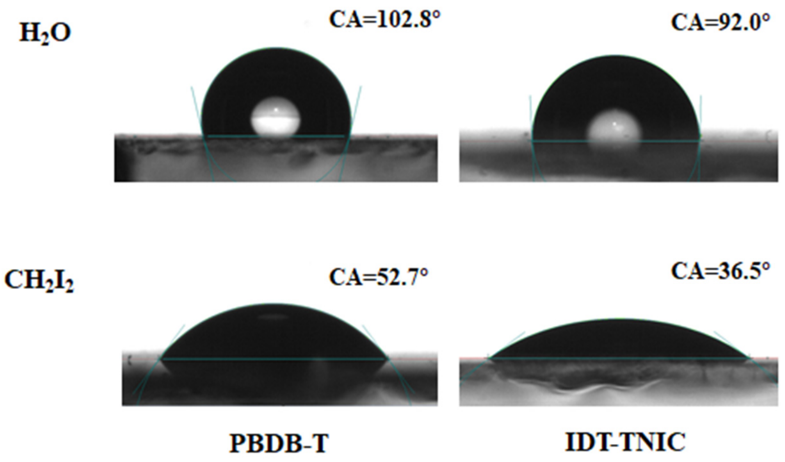

Figure S9. Contact angle images of neat PBDB-T and **IDT-TNIC** films with water and diiodomethane droplet on top.

Table S8. Summary of surface free energy and soluble parameters of neat PBDB-T and **IDT-TNIC** films calculated from contact angles measurement.

**Donor**	γ_{Donor} **(mN m^{-1})**	**Acceptor**	$\gamma_{Acceptor}$ **(mN m^{-1})**	$\chi^{\mathrm{D\text{-}A}}$
PBDB-T	34.33	**IDT-TNIC**	43.66	0.56 [a)]

[a)] χ value was calculated according to Flory–Huggins model of $\chi = (\sqrt{\gamma^D} - \sqrt{\gamma^A})^2$ [4,5].

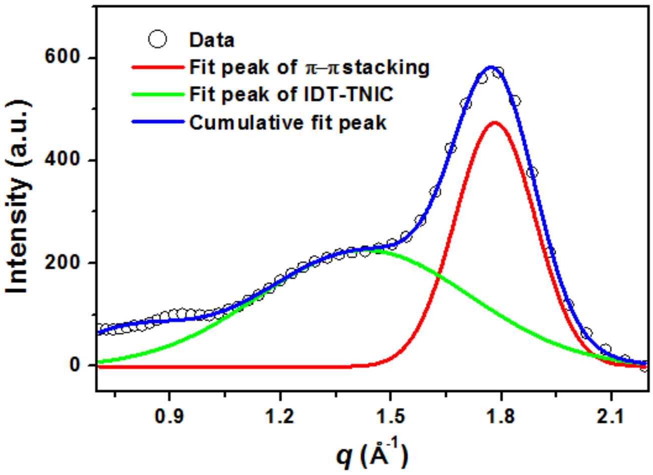

Figure S10. An example of Gaussian fitting for out-of-plane cut. The π-π stacking distance and the FWHM of the π-π stacking peak (010) were obtained from the fitting.

Table S9. GIWAXS test performance parameters of **IDT-TNIC** pure film and PBDB-T: **IDT-TNIC** blend film under optimal conditions

	in plane (100)				out of plane (010)			
	location (Å^{-1})	FWHM	*CL* (Å)	*d*-space (Å)	location (Å^{-1})	FWHM	*CL* (Å)	*d*-space (Å)

**IDT-TNIC**	0.31	0.15	37.68	20.26	1.78	0.25	22.61	3.53
PBDB-T: **IDT-TNIC**	0.30	0.04	141.3	20.93	1.77	0.26	21.74	3.55

Reference

[1] Demadrille, R.; Egreve, S. (FR); Kervella, Y.; Verand, S. (FR). Organic colourant and uses thereof in photovoltaic cells. 〔P〕. US 2014/0290748 A l. 2014-10-02.

[2] Lin, J.; Guo, Q.; Liu, Q.; Lv, J.; Liang, H.; Wang, Y.; Zhu, L.; Liu, F.; Guo, X.; Zhang, M. A noncovalently fused-ring asymmetric electron acceptor enables efficient organic solar cells. *Chin. J. Chem*. **2021**, *39*, 2685–2691. https://doi.org/10.1002/cjoc.202100323.

[3] Li, R.; Liu, G.; Xiao, M.; Yang, X.; Liu, X.; Wang, Z.; Ying, L.; Huang, F.; Cao, Y. Non-fullerene acceptors based on fused-ring oligomers for efficient polymer solar cells via complementary light-absorption. *J. Mater. Chem. A* **2017**, *5*, 23926–23936. https://doi.org/10.1039/C7TA06631G.

[4] Leman, D.; Kelly, M.A.; Ness, S.; Engmann, S.; Herzing, A.; Snyder, C.; Ro, H.W.; Kline, R.J.; DeLongchamp, D.M.; Richter, L.J. In situ characterization of polymer−fullerene bilayer stability, *Macromolecules* **2015**, *48*, 383–392. https://doi.org/10.1021/ma5021227.

[5] Xu, X.; Li, Y.; Peng, Q. Recent advances in morphology optimizations towards highly efficient ternary organic solar cells, *Nano Select* **2020**, *1*, 30–58. https://doi.org/10.1002/nano.202000012.
